# The performance of a lateral flow SARS-CoV-2 antibody assay and semi-autonomous SARS-CoV-2 antisense and sense RNA fluorescence in situ hybridization assay in a prospective cohort pilot study within a Dutch military population

**DOI:** 10.1371/journal.pone.0309091

**Published:** 2024-12-31

**Authors:** Inge D. Wijnberg, Anton J. Soons, Johan G. Reimerink, Marit Wiersma, Marie Christine J. Plat, Tom van Gool, Gijsbert J. Jansen, Cornelis Stijnis, Jack G. Koning, Adam Meijer

**Affiliations:** 1 Expertise Centre, (Micro-) Biology and Infectious Diseases Cluster, Coordination Centre for Expertise on Working Conditions and Health (CEAG), Ministry of Defence, Doorn, NLD; 2 Faculty of Veterinary Medicine, Clinical Sciences, Utrecht University, Utrecht, NLD; 3 Medical Staff, Royal Netherlands Army, Utrecht, NLD; 4 Biotrack, Leeuwarden, NLD; 5 Expertise Centre, Force Health Protection Cluster, Coordination Centre for Expertise on Working Conditions and Health (CEAG), Ministry of Defence, Doorn, NLD; 6 Department of Microbiology/Parasitology, Amsterdam UMC, Amsterdam, NLD; 7 Department of Internal medicine/Centre of Tropical and Travel Medicine, Amsterdam UMC, Location AMC, Amsterdam, NLD; 8 400 Medical Battalion, Royal Netherlands Army, Ermelo, NLD; 9 National Institute for Public Health and the Environment (RIVM), Centre for Infectious Diseases Research, Diagnostics and Laboratory Surveillance, Bilthoven, NLD; Federal Medical Centre Abeokuta, NIGERIA

## Abstract

At the beginning of the COVID-19 pandemic, diagnostic testing was not accessible for mildly ill or asymptomatic individuals. Military operational circumstances exclude the usage of reference laboratory tests. For that reason, at the beginning of the pandemic alternative test methods were needed in order to gain insight into the SARS-CoV-2 status of military personnel. The objectives of this study are to assess whether SARS-CoV-2 antibody rapid lateral flow assay (LFA) in combination with semi-autonomous SARS-CoV-2 antisense and sense genomic RNA fluorescence *in situ* hybridization (FISH) could establish disease status in military personnel in a fieldable setting, and to assess how this combination performed and to determine which type of sample performed best. A proof of concept sub-study regarding the SARS-CoV-2 application of the fieldable Biotrack-MED^®^ FISH analyzer, a semi-autonomous multi-sample filter cytometer, preceded this observational prospective cohort pilot study. Dutch military personnel were included in the 26 June 2020–11 May 2021 period. Blood, nasopharyngeal and oropharyngeal swabs and saliva were tested at days 0 and 14. SPSS version 25 descriptive statistics and Cohen’s kappa assessed agreement between test methods. Both the sensitivity and specificity of the field tests were calculated with ELISA and PCR as reference. Saliva appeared to be the preferred sample type for FISH, where blood was not useful. FISH analysis and LFA results had a concordance of 42% for testing negative, 30% for recovered from infection, 22% for ongoing—and 58% for acute infection in a reference laboratory lab result outcome (RT-PCR or ELISA respectively). The LFA results on serum and full blood corresponded with the ELISA-obtained results (kappa of 0.61 and 0.63 respectively at day 0 and 0.81 and 0.77 respectively at day 14). LFA (full blood-serum), FISH and RT-PCR on saliva did not reach the 90% sensitivity level advised by WHO, with 64–54, 38 and 71% at day 0 and 80–79, 53 and 24% at day 14 respectively.

## Introduction

During the SARS-CoV-2 pandemic, identifying individuals with a SARS-CoV-2 infection was one of the key strategies for deciding on mitigating measures in order to reduce the impact of the disease [1, 2]. During the start of the outbreak in the Netherlands, diagnostic testing was stretched to capacity. Testing was therefore targeted at symptomatic individuals fitting a strict case definition. Diagnostic or preventive testing was not yet available for persons with no or mild (and therefore not fulfilling the case definition) health complaints. Antigen lateral flow assays and molecular point of care tests were not available or had not yet been validated [3].

A specific military need was to identify the potential risk of 1) capacity reduction regarding personnel readiness, 2) combat effectiveness reduction resulting from disease due to SARS-CoV-2 infection and 3) increasing the risk of the spread of SARS-CoV-2 due to asymptomatic infection in a relatively healthy and fit population. Information on the presence of infection within a military population was vital in order to make risk assessments while operating during a COVID-19 pandemic situation at a time when details on (long- and short-term) health effects were unclear. In this phase of the pandemic, vaccines were unavailable and military personnel could not always avoid the risk of exposure to SARS-CoV-2-infected individuals. Because of restricted access to normal test facilities during operations, robust (e.g. transport- and ship-related vibration proof) and reliable tests were needed, fieldable under military operational circumstances. The requirements were as follows: they should remain functional in the hands of operators under (stand-off) supervision of laboratory personnel during variable (weather) conditions; sampling should be minimally invasive; test results should be reliable, regardless of the surrounding temperature, and the logistic and ecological footprint should be minimal [4, 5].

RT-qPCR was the first and the most commonly used test to detect viral RNA, including that of SARS-CoV-2 [6, 7]. Antibodies are most reliably detected in serum using ELISAs, but it takes time to be produced following infection, and they are thus less suitable for early diagnostics. Antibodies can be used to determine past or ongoing infection [8]. However, both of these reference methods are not fieldable and are not suitable for use by non-laboratory personnel. Antibody LFAs are widely used for in-field situations. However, at the start of the SARS-CoV-2 pandemic LFAs were only sporadically available or had barely been evaluated in asymptomatic individuals [9, 10].

Studies on the performance of the fluorescent *in situ* hybridization (FISH) technique as a robust alternative to PCR are relatively scarce [11–14]. Whereas PCR requires the presence of virus RNA or DNA in a sample independent of the presence of cells, FISH by nature (*in situ*) requires the presence of cells in which virus RNA or DNA is present [13, 15–17]. FISH is acknowledged as a useful and cheap alternative diagnostic tool to use in low resource circumstances with the potential to diagnose warfare agents [12]–making it an attractive alternative for military diagnostics-or if clinical case definition based on symptoms is not specific enough. The combination of FISH, enabling the detection of SARS-CoV-2 sense and antisense genomic RNA and sense subgenomic RNA, with the simultaneous use of LFA for antibody detection of COVID-19, might potentially provide useful information on personnel readiness under circumstances where laboratory resources are limited and the infection status of military personnel needs to be identified.

The aims of this study were 1) to test the described diagnostic FISH method using the Biotrack-MED analyzer^®^ on blood, saliva and naso- and oropharyngeal swabs for its potential to identify acute, ongoing or absence of infection with SARS-CoV-2. 2) To test the field performance of an LFA for SARS-CoV-2 antibodies in serum and full blood in a non- or mildly symptomatic military population to identify ongoing or past infection. 3) To determine which sample type performed the best in a military setting and thus to identify the most suitable sample type to be used in a military setting. It was thought that both SARS-CoV-2 genomic material and antibodies tested in a blood sample obtained by one finger prick would be ideal in a military setting. Alternatively, saliva that can be obtained non-invasively by non-laboratory personnel, while presenting fewer health risks, might be preferred to naso- and oropharyngeal swabs in an operational setting [18, 19]. 4) To test whether the Biotrack-MED analyzer^®^ could be a potential diagnostic alternative in operational military settings.

## Methods

### Ethics

The study was approved of by the Medical Ethical Test Evaluation Committee CCMO and registered under METC NL74138, protocol number 20–336. Inclusion of saliva as a sample type was approved after an additional request was granted by the METC under the same protocol number. In addition, permission was obtained as required by the Netherlands Ministry of Defence (Military instruction IMGA/13 ref DOSCO 2020015689). All participants took part in the study on the basis of a signed informed consent regarding the use of their archived and fully anonymized samples for research purposes.

### Study design

The study consisted of 2 parts:

1. proof of concept study; in which confirmed SARS-CoV-2 negative (Group 1) and confirmed SARS-CoV-2 positive (Group 2) controls were used for FISH analysis by the Biotrack-MED^®^ analyzer. The resulting images obtained from these two groups were used to train the FISH algorithm to detect SARS-CoV-2 positive samples on the basis of blood analysis. This algorithm was subsequently used to evaluate the samples obtained from groups 3–5 (see point 2). The sample sizes of groups 1 and 2 was limited by the availability of the sample material at the time of the start of the study.
2. the actual field trial; in which three groups (3,4,5) were studied to evaluate the performance of the selected tests and sample types. The three different test groups were studied to identify SARS-CoV-2 infection in military personnel with no or mild complaints on the basis of clinical history at 0, 7 and 14 days post-inclusion, and full blood, serum, naso-oropharyngeal swabs, and saliva at time points 0 and 14 days post-inclusion.

### Study population definitions—Proof of concept

Group 1, the negative control group:

- White blood cell concentrates for SARS-CoV-2 FISH from 10 individuals that were exposed to avian influenza virus between 01-03-2018 and 30-04-2018 (METC NL13529.041.06). As these dates are well before the COVID-19 pandemic, they were supposed to be negative for SARS-CoV-2 RNA presence;
- SARS-CoV-2 negativity was confirmed by performing SARS-CoV-2 ELISA, human coronaviruses and SARS-CoV-2 protein microarray analysis and SARS-CoV-2 viral neutralization tests on sera of these individuals;
- Other past respiratory viral infections in this group should be well represented, including the common human seasonal coronaviruses. This was confirmed by positivity for these viruses in the protein microarray. The individuals were negative for antibodies against SARS-CoV-1 and MERS coronavirus.

Group 2, the positive control group:

- Venous EDTA blood for SARS-CoV-2 FISH prospectively collected from 42 persons enrolled in a household transmission study between 28-03-2020 and 25-05-2020 (METC NL13529.041.06) that were tested RT-PCR positive for SARS-CoV-2 [20, 21];
- The patients had minor to severe clinical COVID-19 signs;
- The date of the first clinical signs was documented;
- The date of first sampling in relation to the first day of documented illness was documented;
- Due to the timing of the current study and the household studies, mostly only late time point samples of the household study could be collected, as few new patients were included when the current study started.

### Study population definitions—Field trial

Group 3, potentially infected military personnel without clinical signs, was defined as:

- Participants in close contact with an RT-PCR proven SARS-CoV-2 positive-individual;
- At time of inclusion in the study, no clinical signs had been reported by the participants;
- Participants were in close contact with this SARS-CoV-2 positive individual either during the period that this individual exhibited clinical signs or in the three days before this individual started exhibiting clinical signs;
- Close contact was defined as: a minimum of one working day of repeated contact within a zone of 1.5 metres;
- The date of sampling of the SARS-CoV-2 RT-PCR positive sample of the SARS-CoV-2 positive individual was known, as well as the dates during which the SARS-CoV-2 positive individual showed clinical signs.

Group 4, potentially infected military personnel with clinical signs, was defined as:

- Participants in close contact with an RT-PCR proven SARS-CoV-2 positive individual;
- Participants showed COVID-19 clinical signs on day of inclusion in the study;
- Participants were in close contact with this SARS-CoV-2 positive individual either during the period that this individual exhibited clinical signs or in the three days before this individual started exhibiting clinical signs;
- Close contact was defined as: a minimum of one working day of repeated contact within a zone of 1.5 metres;
- The date of sampling of the SARS-CoV-2 RT-PCR positive sample of the SARS-CoV-2 positive individual was known, as well as the dates during which the SARS-CoV-2 positive individual showed clinical signs.

Group 5, military personnel in critical positions:

- Military personnel participating in a critical military process wanted to know their status of infection and in whom SARS-CoV-2 infection would lead to a severe impact on the capability of the military unit or the performance of the assignment;
- At the moment of inclusion there was no known contact with SARS-CoV-2 RT-PCR confirmed infected persons;
- Participants exhibited COVID-19 clinical signs, but without confirmed exposure.

### Recruitment of the participants

For recruitment of participants in groups 3, 4 and 5, military personnel were informed verbally, digitally and/or by telephone about the possibility of participating on a voluntary basis in this prospective descriptive pilot study. Personnel that decided to participate provided written consent. Symptomatic and asymptomatic persons were invited to participate in the 26-06-2020 to 25-05-2021 period. The study was closed on 25-05-2021, since at that time point vaccination campaigns had started. Vaccinations were likely interfere with the results of the study. Participants entering the study were assigned to the defined groups by author AS and were to remain in this assigned group regardless of the development of clinical signs.

### Clinical signs and scores

Signs of COVID-19 were defined according to the Dutch government’s list of clinical signs for COVID-19: loss of–or change in–the normal sense of taste or smell, a new and continuous cough, a rise in core temperature (>37.5°°C), sore throat, rhinitis, sneezing, shortness of breath, dyspnea, headache, nausea, fatigue, and muscle ache [22–25]. Patient histories were recorded at days 0, 7 and 14 on the basis of a standardized questionnaire completed by the participants. A total of 14 reported COVID-19 clinical signs (cough, sore throat, rhinitis, sneezing, shortness of breath, dyspnea, headache, fatigue, muscle pain, joint pain, diarrhea, nausea, abdominal pain, anosmia / ageusia) and were subjectively scored by the participants on a scale of 1 to 5 (1 = absent, 2 = minor, 3 = mild, 4 = moderate, 5 = severe). If a participant reported a fever, the body temperature was reported and scored on a scale of 1 to 5 (1 = no fever, 2 = 37–38°C, 3 = 38–39°C, 4 = 39–40°C, 5 = >40°C).

The clinical complaints per participant per time point (days 0, 7 and 14) were subsequently scored by the data analyst to enable statistical analysis. Persons who failed to answer more than one question on day 0 were reported as having no complaints and received the value 1.

Persons who failed to report clinical signs were assumed to have no complaints and were given the score 1 for the sum calculation. This scoring system resulted in a minimum sum score of 15 for a person without complaints. A sum score of ≥16 indicated a person with complaints. A total score resulted from the questionnaire for days 0, 7 and 14. Clinical signs were defined as “mild” (sum score = 16–18) or “moderate/severe symptoms” (sum score ≥19). The cut-off value for these two categories was based on the median sum score, which was 18.

### Sample collection

Members of the armed forces formed teams responsible for sample collection. They used standard operating instructions, detailing how to collect, transport and process the nasopharyngeal swabs (NP) and oropharyngeal swabs (OP), blood (EDTA and clotting) and oral fluid (saliva) samples. Instructions were given to ensure no sputum was produced instead. At day 0 and day 14, team members collected 12 ml blood per participant by vena puncture, divided in 2 clotting tubes and 1 EDTA tube. Two NP swabs from different nostrils and two OP swabs were taken. In addition, two samples of 3 ml saliva were collected. At the start of the study design in March 2020, saliva had not yet been considered a sample type option. Saliva collection started on 13.07.2020 after additional METC approval.

### Sample processing

Nasopharyngeal swabs (NP) and oropharyngeal swabs (OP) were collected separately and placed combined in two pairs of NP and OP in gelatin-lactalbumin-yeast (GLY) viral transport medium (Mediaproducts BV, Groningen, Netherlands). Saliva was collected in a sterile 15 mL tube. All materials were transported within 6 hours to the different participating laboratories according to the national instructions for the transport, storage and processing of medical samples (see for details, https://www.rivm.nl/documenten/afnametechniek-diagnostiek-seizoensinfluenza-peilstationsurveillance-2021, https://www.sickkids.ca/en/care-services/for-health-care-providers/lab-tests/736-Fluorescence-in-situ-Hybridization-FISH—Blood, https://www.erasmusmc.nl/nl-nl/patientenzorg/laboratoriumspecialismen/klinische-genetica). All samples were transported to 400 Medical Battalion’s laboratory in Ermelo, Netherlands, in an isolated transport box to guarantee room temperature conditions (15-25°C). From this laboratory, samples were distributed to the other participating laboratories: of each person one combined NP/OP swabs sample, a serum sample and a saliva sample were transported in a climate-controlled sample box (4°C) by courier to the National Institute for Public Health and the Environment (RIVM) for RT-qPCR (combined NP/OP sample and saliva sample) and for ELISA antibody detection assay (serum sample). The second serum sample was transported within 6 hours in a similar manner to Amsterdam UMC (AUMC) for LFA antibody detection analyses. Whole blood samples (collected in EDTA tubes) were firstly used for LFA antibody detection analysis directly after arrival in 400 Medical Battalion’s laboratory (at the latest 6 hours after sampling). The second combined NP and OP swabs sample, the second saliva sample and the remainder of the whole blood sample were processed at 400 Medical Battalion’s laboratory in Ermelo for FISH analysis by using a sense SARS-CoV-2 specific probe. The samples were fixated upon arrival as described below and stored at 4° Celsius. An aliquot of the fixated saliva sample was transported in an isolated transport box, to guarantee room temperature conditions (15-25°C), to Biotrack, Leeuwarden, for confirmatory FISH analysis by using the sense and antisense SARS-CoV-2 specific probes. The remainder of samples were stored frozen at -80°C at both AUMC and RIVM in accordance with METC NL74138, protocol number 20–336. The remainder of samples used at Ermelo and Biotrack were destroyed at the end of the study.

### Molecular diagnostics

#### RT-PCR in RIVM reference laboratory

Total nucleic acid was extracted from combined NP and OP swabs and saliva using a Roche MagNApure 96 (MP96) with MagNA Pure 96 DNA and Viral NA Small Volume Kit (Roche). A two-hundred μl sample was mixed with 275 μl MP96 lysis buffer, including equine arteritis virus (EAV) internal control and yeast tRNA stabilizer. The remainder of the samples were stored in a frozen state at -80°C. Total nucleic acid was eluted in 50 μl Tris- EDTA buffer. RT-qPCR was performed on 5 μl total nucleic acid using TaqMan® Fast Virus 1-Step Master Mix (Thermo Fisher) on a Roche LC480 II thermal cycler with SARS-like beta coronavirus (Sarbeco) specific E-gene primers and probe and EAV primers and probe as described previously [6, 26]. As no other Sarbeco viruses were endemic or circulating at the time, a positive Sarbeco E-gene RT-qPCR was validly taken as positive for SARS-CoV-2. All samples were also tested with the RdRp gene primers and probe slightly modified from those published by Corman et al. 2020 [6] in order to become SARS-CoV-2 specific and of equal analytical sensitivity as the E-gene RT-qPCR. The modified primers and probe were: RdRp_SARS-F2 GTGAAATGGTCATGTGTGGCGG; RdRp_SARS-R2 CAAATGTTAAAAACACTATTAGCATAAGCA; RdRp_SARS-P2.2 CCAGGTGGAACCTCATCAGGAGATGC. Both RT-qPCR assays amplify positive sense genomic RNA found in infected cells and virus particles and the negative sense genomic intermediate RNA found in infected cells only. In addition, the E-gene RT-qPCR assay amplifies sub-genomic messenger RNA found in infected cells only. A sample was considered positive for SARS-CoV-2 if the E-gene RT-qPCR was positive and/or the RdRp-gene RT-qPCR was positive. A patient was considered RT-qPCR positive for SARS-CoV-2 if either or both sample types, combined NP and OP swabs and saliva, were positive in RT-qPCR.

#### Fluorescence in situ hybridization (FISH)

*The probes*. The fluorescent probes utilized were directed at the nucleocapsid gene of SARS-CoV-2 and comprised of 1) a Cy-3 labeled antisense genomic RNA-specific probe (Biotrack COV19 Probe, positive, sense orientation, 24 nucleotides, GC: 41.7%, Tm: 60.2°C) and 2) its complementary sequence, a Cy-3 labeled sense genomic RNA-specific probe (Biotrack COV19 GProbe, negative, antisense orientation, 24 nucleotides, GC: 41.7%, Tm: 60.2°C). The COV19 Probe stained the antisense replicative form of the virus genome and the COV19 GProbe the sense oriented virus RNA genome and subgenomic mRNAs. Both probes have an *in silico* specificity of 100% and have been used in SARS-CoV-2 patient studies [17, 27].

*FISH on saliva samples*. As soon as it became clear from the literature that saliva could be a potentially useful sample, METC permission was sought and granted to include saliva samples in the study. To perform the FISH procedure, 3 ml saliva was fixed for 10 minutes by diluting 1:1 (v/v) and mixing the saliva with the same volume of methanol. This fixed cell suspension was mixed 1:2 (v:v) with either the COV19 Probe or the COV19 GProbe in Biotrack hybridization buffer (NaCl, Tris-HCl, Triton X-100, SDS, the exact concentrations are a trade secret). The mixture was hybridized at 50°C for 1.5 hours under a dark conditions. Thereafter, 1 ml pre-warmed (60°C) Biotrack Wash Buffer (NaCl, Tris-HCl, the exact concentrations are a trade secret) was added for 30 minutes at 60°C under dark conditions dark. Subsequently, the suspension was centrifuged (10 min. at 800 x g), the supernatant removed and the pellet suspended with 50 μl Milli-Q.

From the saliva samples hybridized with the COV19 Probe, 30 μl was spotted onto a plastic sample container (a filter cup), which was fitted into a Biotrack analysis disk. The sample was dried for 25 minutes at 50°C and placed into the Biotrack-MED^®^ analyzer for autonomous microscopic reading in the military field laboratory.

From the saliva samples hybridized with the COV19 GProbe, 10 μl was spotted onto a microscopic slide with 10 reaction wells (VWR, Netherlands), dried for 10 minutes at 50°C and mounted with Fluoroshield (VWR, Netherlands) and evaluated in multiple planes. As the application of the Biotrack-MED® analyzer had not (yet) been trained for the recognition of COV19 GProbe positive cells during this phase of the study, the images generated by the Biotrack-MED^®^ analyzer were blinded and manually analyzed independently by two experienced researchers at the Biotrack company in Leeuwarden, the Netherlands, and presented as a positive or negative result.

*FISH on blood samples*. For the FISH procedure on whole blood, a Biotrack proprietary test kit (BTMED COV19 test kit) was used, consisting of the Biotrack COV19 Probe in the Biotrack hybridization buffer, Biotrack Wash Buffer and filter cups. The COV19 Gprobe was not yet available at the time of whole blood analysis and these blood samples were not suitable for long term storage for later use. Blood was collected by vena puncture and stored in EDTA as described above and the sample was homogenized before use. 117 μl blood was added to 1312 μl Milli-Q, which was incubated at room temperature for 12 seconds, while in-and-out pipetting to hemolyze the erythrocytes. After 12 seconds, 76 μl 10% (w/v) NaCl solution was added and centrifuged for 10 minutes at 800 x g with soft acceleration and deceleration. The supernatant was removed and the pellet resuspended in 15 μl 0.9% (w/v) NaCl solution; 500 μl methanol was added, cells resuspended and incubated for 10 minutes at room temperature. Following this, the sample was centrifuged for 10 minutes at 800 x g, where after the supernatant was removed and the remaining methanol was left to evaporate for 20 minutes at 50°C. 50 μl of the Biotrack COV19 Probe was added to the pellet, cells resuspended and incubated for 1.5 hours at 50°C in a dark room. Thereafter, 1 ml pre-warmed Biotrack Wash Buffer was added, cells resuspended an incubated for 30 minutes at 50°C under dark conditions. Subsequently, the suspension was centrifuged (10 minutes at 800 x g), the supernatant removed and the pellet resuspended with 30 μl 0.9% (w/v) NaCl solution. Finally, 10 μl was spotted onto a plastic sample container (a filter cup), which was fitted into a Biotrack analysis disk. The sample was dried for 15 minutes at 50°C and put into the Biotrack-MED^®^ analyzer placed at a military field laboratory for autonomous microscopic reading.

*FISH on NP/OP swabs*. Cell quality and concentration in test groups appeared too low, as after 130 tested samples almost 40% did not provide reliable cytology results. It was thus concluded that NP/OP swabs were not useful for further processing and FISH analysis. For that reason, the results are not further described here.

*FISH data acquisition and interpretation—proof of concept*. The artificial intelligence (AI) component of the Biotrack-MED analyzer^®^ was trained to recognize and interpret the generated microscopic images of blood and saliva. AI training regarding blood samples was achieved by utilizing a known negative and a known positive control group (Group 1 and Group 2, respectively) using the COV19 probe. Images generated by the SARS-CoV-2 application were analyzed by three experienced researchers in order to obtain data about the morphometric parameters of negative and positive white blood cells in blood, after which the parameters were added to the computer algorithm.

The AI training regarding saliva was simultaneously achieved in another study, as described by Tamminga et al [27]: images of 10 known RT-PCR SARS-CoV-2 positive and 18 known RT-PCR SARS-CoV-2 negative persons (age 18–70, 88% men, 12% women) were utilized to determine the morphometric parameters of negative and positive white blood cells in saliva. This was done by three experienced researchers, after which the parameters were added to the computer algorithm. Epithelial cells (and a positive FISH response in these cells) could be seen occasionally, but were ignored in the analysis. Before the computer algorithm was finished, the samples were first blinded and manually analyzed independently by three experienced researchers. After the computer algorithm was finished, AI analysis was performed on all samples.

COV19 GProbe fluorescently labeled saliva samples from groups 3–5 were, at this stage of the R&D track, manually analyzed by utilizing a Leica DM2500 fluorescence microscope equipped with a Leica EL6000 mercury lamp and a Leica DFC450C camera. The samples were blinded and manually analyzed at a magnification of 400x. For quality control purposes, this analyses was performed by two experts in FISH analysis. These researchers were blinded with regard to each other’s interpretations.

*FISH data acquisition and interpretation—field trial*. The fluorescently labeled saliva and blood samples with the COV19 Probe were read and documented utilizing the Biotrack-MED^®^ analyzer (patent EP 08874964.3, CE-IVD: NL-CA002-2020-51055), a semi-autonomous, multi-sample filter cytometer (Biotrack, Leeuwarden, Netherlands). The Biotrack-MED^®^ analyzer contains a multi-layer neural network that is trained for the interpretation of FISH-based images utilizing the SARS-CoV-2-specific COV19 probe, known as the SARS-CoV-2 application. The SARS-CoV-2 application produced approximately 150 microscopic fluorescence images (in Z-direction) at a magnification of 200x from which visually recognizable objects were isolated by a computer vision algorithm. Morphometric parameters (n = 20, including shape, area, roundness, brightness and number of spots) were calculated per object. The morphometric parameter scores were subsequently entered into the neural network of the SARS-CoV-2 application, resulting in an autonomously generated test result for the presence of SARS-CoV-2 RNA (positive or negative). For this R&D study, blinded resulted microscopic fluorescent images were checked by two experts in FISH analysis. Their results were compared with the results obtained by the Biotrack-MED^®^ analyzer at the field lab for quality control reasons. When the results between the two experienced researchers and the Biotrack-MED^®^ analyzer did not match, a third researcher with experience in this technique also examined the microscopic fluorescent images in order to make the final decision. Interpretation was qualitative: a positive or negative result was reported.

### Serological diagnostics

#### ELISA analysis on serum in RIVM reference laboratory

The Wantai SARS-CoV-2 total antibody ELISA (Beijing Wantai Biological Pharmacy Enterprise, Beijing, China; catalogue number WS1096) was performed on serum in accordance with the manufacturer’s instructions. This assay is a two-step incubation sandwich ELISA using the recombinant receptor-binding domain of the SARS-CoV-2 spike protein as antigen for the detection of total antibodies against SARS-CoV-2. Optical density (OD) is measured at 450 nm and the antibody OD ratio for each sample is calculated as the ratio of the OD of a particular sample in relation to the reading of a calibrator included in the kit. A ratio of ≥1.0 was considered positive for the presence of SARS-CoV-2 specific antibodies, as per the manufacturer’s instructions. This kit is validated for the qualitative, not quantitative, interpretation of the test results, although around sero-conversion an increase in signal can be observed until saturation.

#### Lateral flow diagnostics on serum

The COVID-19 IgG/IgM rapid test cassette (Biozek medical, catalogue number BNCP-402) was performed on serum in accordance with the manufacturer’s instructions at the diagnostic laboratory of the AUMC. This assay is a qualitative membrane-based immunoassay for the detection of Nucleocapsid IgG/IgM antibodies and Receptor Binding Domain (RBD) IgG/IgM antibodies against COVID-19 in whole blood, serum or plasma specimens. Only IgG results were provided after visual analysis and interpretation by AUMC laboratory personnel in triplicate. Interpretation was qualitative: a positive or negative result was reported. Results of the LFA were considered positive if the IgG band was reactive.

#### Lateral flow analysis on full blood

The COVID-19 IgG/IgM rapid test cassette (Biozek medical, catalogue number BNCP-402) was also performed on EDTA whole blood in accordance with the manufacturer’s instructions in the military field laboratory. The results were visually analyzed and interpreted by two of the battalion laboratory’s technicians. Interpretation was qualitative: a positive or negative result was reported. Results of the LFA were considered positive if the IgG, the IgM band or both were reactive. Results for IgG and IgM were reported separately.

#### Interpretation of test results

Nucleic acid and antibody detection results were used to categorize participants in groups: SARS-CoV-2 negative, acute infection, ongoing infection and past infection. The definitions are described in Table 1.

**Table 1:** Definitions of interpretation of the status of infection, based on combinations of results of RT-PCR with E, or a combination of FISH with LFA.

	RT-PCR FISH Day 0 +	RT-PCR FISH Day 14 +	RT-PCR FISH Day 0 -	RT-PCR FISH Day14 -	E LFA Day 0 +	E LFA Day14 +	E LFA Day 0 -	E LFA Day 14 -
**Negative**			y	y			y	y
**Acute infection**	y	y/n			n	y		
					y	y, E ≥4 x increase		
**Ongoing infection**	y	y			Y, high or at saturation	y, E <4 x increase or at saturation		
**Past infection**			y	y	y	y		

+: positive result; -:negative result: y: yes; n: no; RT-PCR: reverse transcription polymerase chain reaction; E: ELISA, FISH: fluorescence *in situ* hybridization; LFA: lateral flow assay.

### Statistical analyses

#### Power analysis

The sample size of the trial was predefined by power analysis calculations (Table 2). The sample size was determined using the nQuery calculator and formula of Daniel, 1999 [28], and was based on an estimated prevalence and 90% sensitivity and specificity (at the time, the criteria set by WHO regarding useful COVID-19 diagnostic tests) [3].

**Table 2:** Power analysis calculation.

Group	Estimated prevalence	confidence interval	1 or 2 sided	Sens	Spec	Target confidence interval width	Calculated sample per group
3	0.6	0.95	2	0.9	0.9	0.3	53
4	0.8	0.95	2	0.9	0.9	0.3	106
5	0.1	0.95	2	0.9	0.9	0.3	210

Sens: sensitivity; Spec: specificity.

### Statistics

Descriptive statistics using SPSS 25 were used to present baseline characteristics, outcomes of test results at day 0 and day 14, and the reported clinical signs at day 0, day 7, and day 14.

For all test groups, logistic regression analyses were conducted to assess whether reported clinical signs at day 0 were associated with a positive test result. Therefore, a variable containing three categories was established. The category “no clinical signs” (sum score = 15) was used as reference category. Furthermore, two categories of clinical signs were defined: “mild” (sum score = 16–18) and “moderate/severe symptoms” (sum score ≥19). The cut-off value for these two categories was based on the median sum score, which was 18. A value is considered significant if the reference 1 is not included in the CI interval. A second model was used to determine the confounding factors “gender” and “age”.

*Kappa correlations were calculated between tests*, *samples and timepoints*. Cohen’s kappa was calculated because it measures the extent of agreement between the evaluations of two raters when both are rating the same object. A value of 1 indicates perfect agreement. A value of 0 indicates that agreement is no better than chance. A kappa value >0.8 is considered good, a kappa of > 0.6 as reasonable. This scale of interpretation was previously defined by Warrens and co-workers [29]. Correlations were calculated to assess agreement for FISH test results with the RT-PCR-swab and RT-PCR-saliva results at day 0 and day 14. The same was calculated for ELISA compared with LFA results. These analyses were performed separately for Group 3, Group 4, Group 5, and in total. In addition, the clinical scores, RT-PCR on NP/OP swabs in combination with ELISA results, were interpreted by fourauthors (CS, AJS, IDW, AM) in order to categorize each individual for: no, acute, ongoing, or past infection with SARS-COV-2 (Table 1). This assessment was repeated using FISH on saliva and/or blood and LFA test results (Table 1). Author AM validated the RT-PCR and ELISA results. TG validated the LFA serum results (pos/neg). GJ validated the FISH results (pos/neg). AS and JK validated the LFA full blood results (pos/neg). The outcome of interpretation of both test sets (field tests versus gold standard testing) were compared with each other using Cohen’s kappa correlations and cross tabulations to illustrate the results. Sensitivity and specificity of FISH, LFA and RT-PCR on saliva samples were calculated at days 0 and 14 with RT-PCR on NP/OP swabs and ELISA as reference. The interpretation of sensitivity and specificity scores was based on the recent insights of Schreffler and co-workers [30].

## Results

### Proof of the concept—FISH analysis

From the available negative control samples in Group 1, 13 white blood cell samples were available for FISH analysis. After FISH analysis, the white blood cell concentrates analysis of these 13 isolates resulted in negatively interpreted results. This indicated no presence of SARS-CoV-2 in the samples at the moment of sampling, which was as expected as these samples were collected well before the SARS-CoV-2 pandemic. Antibody tests confirmed that these individuals did not have a past infection with SARS-CoV-2 and had previous infections with seasonal coronaviruses. These images could therefore be used as SARS-CoV-2 negative references for training the FISH algorithm.

The participants in Group 2 were tested as part of an ongoing study at the RIVM at three different points in time, after inclusion as SARS-CoV-2 RT-PCR confirmed index patient or household contact [20]. Of the 42 individuals in Group 2, 42 EDTA blood samples were available for FISH analysis and training of the FISH algorithm. Patients were included that had—at any time point—an RT-PCR confirmed infection or antibody confirmed past infection with SARS-CoV-2. However, only at the late third time point [21] (4–6 weeks post inclusion) were whole blood samples available for FISH analysis, resulting in a low number of still NP/OP SARS-CoV-2 RT-PCR positive individuals at the time point the EDTA blood was collected. Of these 42 SARS-CoV-2 positive participants, 36 were tested FISH positive, while only one of the 36 FISH positive individuals was SARS-CoV-2 RT-PCR positive, in an upper airway sample at time point 3 and another one in a feces sample. Feces samples were not included in the study design of the field trial, thus these results were not used for the training of FISH algorithms. However, antibody tests were positive for anti-SARS-CoV-2 antibodies in all except one individual in which only transient weak antibody positivity was detected without any RT-PCR positivity. NP/OP samples contained insufficient cell material, making these unsuitable for FISH analysis. In conclusion, small numbers of positive samples were used for training the FISH algorithm in positive and negative interpretation.

### Field trial–Demographics

Males and the age group of 25–34 were overrepresented in study groups 3–5, as expected in a Dutch military study group (Table 3). The number of participants was highest in the group that wanted to know their status for operational reasons (Group 5). The number of participants were lowest in the group with health complaints that had had close contact with a RT-PCR confirmed SARS-CoV-2 positive person (Group 4).

**Table 3:** Demographic characteristics of included individuals by group and total.

		Total (N = 461)		Group 3 (N = 114)		Group 4 (n = 79)		Group 5 (N = 268)	
		N	%	N	%	N	%	N	%
Gender n = 461	Male	368	83.7	99	86.8	59	74.7	228	85.1
	Female	75	16.3	15	13.2	20	25.3	40	14.9
Age known n = 459	15–24 yrs	127	27.5	38	33.3	36	46.2	53	19.9
	25–34 yrs	173	37.7	51	44.7	17	21.8	105	39.3
	35–44 yrs	65	14.2	13	11.4	12	15.4	40	15.0
	45–54 yrs	74	16.1	11	9.6	13	16.7	50	18.7
	55–69 yrs	20	4.4	1	0.9	0	0	19	7.1

### Field trial test results

Of 66 participants a second complete sample set at day 14 was not available. A relatively low number of military personnel tested positive on RT-PCR at day 0, and this total percentage was slightly lower at day 14 (Table 4). For saliva, this total percentage was lower and the decrease at day 14 was more obvious. An ELISA antibody response was positive at day 0 in 20% of participants. This percentage was similar at day 14. The LFA IgG showed an increase in antibody presence in the included population over time, regardless which blood sample was used, and with a comparable positive percentage at day 14 similar to the ELISA results. Usage of the FISH-COV19 probe on blood and saliva showed fewer positive results than when PCR on NP/OP swab or saliva was used; usage of the FISH-COV19 Gprobe on saliva resulted in more positive outcomes than RT-PCR on NP/OP swab or saliva.

**Table 4:** Test results for COVID-19 in military personnel (N = 461).

	Day 0			Day 14		
Groups 3 through 5 together	N	N positive	% positive	N	N positive	% positive
LFA full blood IgG	461	62	13%	391	80	20%
LFA full blood IgM	461	60	13%	391	55	14%
LFA serum IgG	461	58	13%	391	81	21%
ELISA total Ig	461	92	20%	391	95	21%
FISH_COV19 probe full blood	437	32	7%	378	18	5%
FISH_COV19 probe saliva	420	21	5%	364	11	3%
FISH_COV19G probe saliva	455	119	26%	389	93	24%
RT-PCR-NP/OPswab	455	57	13%	395	42	11%
RT-PCR_saliva	452	46	10%	395	20	5%

Ig: immunoglobulin; FISH: fluorescence *in situ* hybridization; LFA: lateral flow assay; N: number, NP: nasopharyngeal; RT-PCR: reverse transcription polymerase chain reaction.

Most positive RT-PCR tests were found on NP/OP swab samples in Group 4, day 0 (46%), followed by saliva samples in this group at day 0 (42%) (Table 5). At day 14 in Group 4, the RT-PCR saliva test was less frequently positive than the NP/OP swab results, whereas the ELISA was, logically, more frequently positive at day 14 for Group 4, but not for groups 3 and 5. The highest number of positive FISH results were seen after using the FISH-COV19 Gprobe on saliva samples in Group 4 at day 0 (30%), followed by Group 3 at day 0 (28%). For ELISA, most positive antibody samples were found in Group 4 at day 14 (56%), followed by Group 4 at day 0 (32%). For the antibody LFA test, most positive tests were found for IgG in full blood and serum also in Group 4 at day 14 (both 50%), followed by IgM in full blood at day 14 in Group 4 (32%).

**Table 5:** Positive test results per group at day 0 and 14.

	RT-PCR NP/OP	RT-PCR Saliva	FISH-COV19G Saliva	FISH-COV19 saliva	FISH-COV19 blood	ELISA Total Ig	LFA IgG Full blood	LFA IgM Full blood	LFA Serum IgG
**Group 3:** potentially infected military personnel, without clinical signs									
Day 0% Positive	**8**	6	28	3	12	**18**	9	14	8
Day 14% Positive	**8**	5	28	3	4	**18**	12	9	11
N D0	**114**	112	114	100	105	**114**	114	114	114
N D14	**111**	111	110	98	105	**111**	111	111	111
**Group 4:** potentially infected military personnel, with clinical signs									
Day 0% Positive	**46**	42	30	4	7	**32**	16	20	18
Day 14% Positive	**39**	18	19	3	7	**56**	50	32	50
N D0	**79**	79	77	68	72	**79**	72	72	72
N D14	**72**	72	72	69	70	**40**	36	26	36
**Group 5:** military personnel in vital positions, no known contact with confirmed SARS-CoV-2 case, with or without clinical signs									
Day 0% Positive	**5**	2	24	6	5	**18**	15	10	13
Day 14% Positive	**2**	1	23	3	4	**17**	15	11	16
N D0	**262**	261	264	252	260	**268**	268	268	268
N D14	**212**	212	207	197	203	**208**	208	208	208

D: Day; FISH: fluorescence *in situ* hybridization; LFA: lateral flow assay; N: number; NP: nasopharyngeal; OP: oropharyngeal; RT-PCR: reverse transcription polymerase chain reaction; Bold: the reference test methods. Numbers in bold indicate the reference test results.

### Field trial—Clinical signs

In total, 297 (67%) persons reported one or more clinical signs on day 0 (Table 6/appendix).

The clinical signs reported the most on day 0 were coughing, rhinitis, sneezing, headache and fatigue.

**Table 6:** Clinical signs at day 0.

		Total		Group 3		Group 4		Group 5	
		N	%	N	%	N	%	N	%
**Fever**	No	441	98%	113	100%	66	92%	262	98%
	Yes	11	2%	0	0%	6	8%	5	2%
**Cough**	No	337	73%	84	74%	32	41%	221	83%
	Yes	122	27%	29	26%	47	59%	46	17%
**Sore throat**	No	394	86%	93	83%	51	65%	250	94%
	Yes	64	14%	20	17%	28	35%	17	6%
**Rhinitis**	No	240	52%	52	46%	23	29%	165	62%
	Yes	219	48%	61	54%	56	71%	102	38%
**Sneezing**	No	326	71%	77	68%	48	61%	201	75%
	Yes	133	29%	36	32%	31	39%	66	25%
**Shortness of breath**	No	419	91%	107	95%	57	72%	255	96%
	Yes	40	9%	7	5%	22	28%	12	4%
**Dyspnoea**	No	430	94%	109	97%	63	80%	258	97%
	Yes	29	6%	4	3%	16	20%	9	3%
**Headache**	No	351	77%	84	74%	42	53%	225	84%
	Yes	108	23%	29	26%	37	47%	42	16%
**Fatique**	No	321	70%	74	66%	39	50%	208	78%
	Yes	137	30%	39	34%	40	50%	59	22%
**Muscle pain**	No	386	84%	90	80%	54	68%	242	91%
	Yes	72	16%	23	20%	25	32%	24	9%
**Joint pain**	No	428	93%	103	93%	65	82%	260	97%
	Yes	31	7%	8	7%	14	18%	7	3%
**Diarrhoea**	No	430	94%	103	91%	68	86%	254	95%
	Yes	29	6%	10	9%	11	14%	13	5%
**Abdominal pain**	No	423	92%	104	92%	71	90%	248	93%
	Yes	36	8%	9	8%	8	10%	19	7%
**Nausea**	No	435	94%	105	94%	70	89%	260	97%
	Yes	23	6%	7	6%	9	11%	7	3%
**Anosmia / ageusia**	No	418	91%	106	95%	51	65%	261	98%
	Yes	40	9%	6	5%	28	35%	6	2%

Excluded are participants who did not fill in the questionnaire or were lost to follow up (day 0: n = 0, day 7: n = 52, day 14: n = 68).

Anosmia/ageusia was reported most frequently in Group 4 (Table 7). As expected, reported signs were the highest in group 4, followed by group 3 and group 5 and the number of persons with reported signs declined over time in all groups (Table 7).

**Table 7:** Presence or absence of clinical signs* at day 0, day 7 and day 14.

		Total		Group 3		Group 4		Group 5	
		N	%	N	%	N	%	N	%
**Day 0**	No signs	148	32%	30	26%	5	6%	113	42%
	With signs **	313	68%	84	74%	74	94%	155	58%
**Day 7**	No signs	172	42%	38	35%	24	35%	110	48%
	With signs	237	58%	72	65%	45	65%	120	52%
**Day 14**	No signs	177	45%	42	38%	25	35%	110	52%
	With signs	216	55%	68	62%	46	65%	102	48%

*Excluded are participants who did not fill in the questionnaire or were lost to follow up (day 0: n = 0, Day 7: n = 52, day 14 n = 68). **with signs = defined as 1 or more signs reported.

### Clinical sign scores

Clinical complaints were scored as mentioned earlier (M&M): the scoring system resulted in a minimum sum score of 15 for a person without complaints. ‘No complaints’ was scored as 1, resulting in presence of complaints if sum scores were ≥16: A sum score of ≥16 indicates a person with complaints. In Group 4, in which highest complaint scores were present (Table 8) the values were highest. Median scores (and Q1-) are presented, since the data did not exhibit a normal distribution. In total, 313 (68%) persons reported one or more clinical signs on day 0 and even Group 3 reported clinical signs by the time the medical staff reviewed the clinical history after inclusion in the study. The number of reported clinical signs was the highest in Group 4, followed by Group 3 and Group 5. The number of persons with reported signs declined over time in all groups.

**Table 8:** Median scores of clinical signs per group at day 0,7, and 14.

		Day 0	Day 7	Day 14
Total	No signs, N (%)	147 (33%)	164 (42%)	169 (44%)
	With signs, N (%)	297 (67%)	230 (58%)	213 (56%)
	Number of clinical signs, Median (Q1—Q3)	3 (1.5–5)	3 (1–4)	3 (1–4)
	Sum score clinical signs, Median (Q1—Q3)	18 (17–21)	18 (16.8–20)	18 (16–20)
**Group 3**	No signs, N (%)	30 (28%)	36 (34%)	38 (37%)
	With signs, N (%)	78 (72%)	69 (66%)	66 (63%)
	Number of clinical signs, Median (Q1—Q3)	3 (1–4)	3 (2–4)	3 (1.8–4)
	Sum score clinical signs, Median (Q1—Q3)	19 (17–20)	18 (17–20)	18 (16.8–19.2)
**Group 4**	No signs, N (%)	5 (7%)	24 (36%)	25 (35%)
	With signs, N (%)	66 (93%)	43 (64%)	46 (65%)
	Number of clinical signs, Median (Q1—Q3)	5 (2.8–7)	4 (2–6)	3 (1–5)
	Sum score clinical signs, Median (Q1—Q3)	21 (18–26.3)	20 (17–24)	19 (17–21)
**Group 5**	No signs, N (%)	112 (42%)	104 (47%)	106 (51%)
	With signs, N (%)	153 (58%)	118 (53%)	101 (49%)
	Number of clinical signs, Median (Q1—Q3)	2 (1–4)	2 (1–3)	2 (1–4)
	Sum score clinical signs, Median (Q1—Q3)	18 (16.5–19)	17 (16–19)	18 (16–19)

Based on these results, apparently all groups showed similar symptoms despite the inclusion criteria for Group 3. Therefore only results from pooled groups are presented in Table 9.

**Table 9:** Relationship between clinical signs at day 0 and test results.

Positive result with indicated technique and sample type at indicated day.	No clinical signs (N = 148)	Mild clinical signs (N = 163)	Moderate/Severe clinical signs (N = 150)
**RT-PCR_NP/OP swab (day 0)**	1	1.90 (0.74–4.86)	**6.33 (2.71–14.75)**
*Cases*	*7*	*14*	*36*
**RT-PCR_saliva (day 0)**	1	1.59 (0.47–5.55)	**10.94 (3.78–31.69)**
*Cases*	*4*	*7*	*35*
**FISH_full blood (day 0)**	1	1.51 (0.57–4.00)	2.14 (0.84–5.48)
*Cases*	*7*	*11*	*14*
**FISH COV19_saliva (day 0)**	1	2.06 (0.70–6.08)	1.02 (0.29–3.62)
*Cases*	*5*	*11*	*5*
**FISH COV19G _saliva (day 0)**	1	0.77 (0.45–1.30)	1.31 (0.79–2.17)
*Cases*	*38*	*34*	*47*
**ELISA ratio (day 14)**	1	1.40 (0.75–2.63)	**2.70 (1.48–4.92)**
*Cases*	*20*	*30*	*45*
**LFA full blood IgG (day 14)**	1	1.35 (0.69–2.65)	**2.55 (1.35–4.82)**
*Cases*	*17*	*25*	*38*
**LFA full blood IgM (day 14)**	1	0.33 (0.15–0.76)	1.15 (0.60–2.17)
*Cases*	*21*	*9*	*25*
**LFA serum IgG (day 14)**	1	1.27 (0.65–2.46)	**2.39 (1.28–4.47)**
*Cases*	*18*	*25*	*38*

Odds ratios are presented with their 95% confidence intervals (CI). Bold indicates a significant result. FISH: fluorescence *in situ* hybridization; LFA: lateral flow assay; N:number; NP: nasopharyngeal; OP: oropharyngeal; RT-PCR: reverse transcription polymerase chain reaction. Clinical signs were defined: “mild” (sum score = 16–18) or “moderate/severe symptoms” (sum score ≥ 19). The cut-off value for these two categories was based on the median sum score, which was 18.

### Relationship between test results and clinical signs

Logistic regression (Table 9) showed that, overall, at day 14 persons with moderate/severe clinical signs were more likely to have a positive RT-PCR or a positive antibody test result (ELISA and LFA IgG), compared to those who reported no symptoms at day 0 (reference day). This relationship was most significant between severe complaints and PCR saliva at day 0 (odds ratio 10.94, 95% confidence interval (CI) 3.78–31.69) and PCR NP/OP swab at day 0 (odds ratio 6.33, 95% CI 2.71–14.75). No significant relationships were shown for clinical signs at day 14 with FISH test results and LFA-IgM on full blood, nor for any of the test results in participants reporting mild clinical signs (Table 9). Adjustments for gender and age did not change the relationships; these data have therefore not been presented in this article.

### Correlations between tests, samples and time points

Kappa correlations were calculated between the results of FISH and PCR on NP/OP swabs and saliva, and LFA antibodies and ELISA at day 0 and at day 14 (Table 10). In addition, Kappa correlations were calculated between PCR NP/OP swabs at day 0 and ELISA at day 14, since it was expected that a positive PCR or FISH test on day 0 will result in an antibody response at day 14 due to the induced immune response following infection (not shown in table). Reasonable to good correlations are highlighted. The results show a reasonable correlation between the LFA-IgG and ELISA on total Ig at day 0 as well as at day 14 (Table 10). Overall good correlations were present between LFA-IgG performed in full blood and serum samples. Good to reasonable correlations were present between PCR performed in NP/OP and saliva samples at day 0, especially in Group 4 but not in Group 5. The LFA for IgG on full blood correlated reasonably to good with the ELISA, but the LFA-IgG on serum correlated less with ELISA, especially in Group 3. None of the FISH tests correlated with the RT-PCR (Table 10). At day 14, the positive PCR results correlated reasonably with a positive ELISA result in Group 4, as apparently in this group the RT-PCR was still positive while a positive antibody response was already present.

**Table 10:** Cohen’s Kappa correlation at day 0 and at day 14 between tests and sample types groups together and per group.

	Day 0 all groups together				Day 14 all groups together			
	LFA serum IgG	RT-PCR NPOP swab	RT_PCR Saliva	ELISA Total Ig	LFA Serum IgG	RT-PCR NPOP swab	RT_PCR Saliva	ELISA Total Ig
LFA full blood IgG	**0.92**			**0.63**	**0.95**			**0.77**
LFA full blood IgM	0.34			0.31	0.33			0.34
LFA serum IgG				**0.61**				**0.81**
FISH full blood		-0.02	-0.002			-0.03	-0.05	
FISH COV19_saliva		0.04	0.07			-0.01	-0.04	
FISH COV19G_ saliva		0.10	0.06			-0.001	0.08	
RT-PCR_NPOP swab			**0.70**				0.52	
	**Day 0 Group 3**				**Day 14 Group 3**			
	LFA serum IgG	RT-PCR NPOP swab	RT-PCR Saliva	ELISA Total Ig	LFA Serum IgG	RT-PCR NPOP swab	RT-PCR Saliva	ELISA Total Ig
LFA full blood IgG	**0.94**			0.52	**0.87**			0.54
LFA full blood IgM	0.24			0.24	0.19			0.24
LFA serum IgG				0.46				0.57
FISH full blood		-0.10	-0.08			0.12	-0.04	
FISH COV19_ saliva		-0.05	-0.04			-0.05	-0.04	
FISH COV19G_ saliva		0.03	-0.06			-0.03	0.04	
RT-PCR_NPOP swab			**0.60**				0.55	
	**Day 0 Group 4**				**Day 14 Group 4**			
	LFA serum IgG	RT-PCR NPOP swab	RT-PCR Saliva	ELISA Total Ig	LFA Serum IgG	RT-PCR NPOP swab	RT-PCR Saliva	ELISA Total Ig
LFA full blood IgG	**0.96**			0.46	**1.00**			**0.89**
LFA full blood IgM	0.51			0.32	0.42			0.33
LFA serum IgG				0.50				**0.89**
FISH full blood		0.04	-0.07			-0.14	-0.12	-0.09
FISH COV19_ saliva		0.10	0.12			0.02	-0.05	
FISH COV19G_saliva		0.28	0.17			0.10	0.23	
RT-PCR_NPOP swab			**0.82**				0.45	
	**Day 0 Group 5**				**Day14 Group 5**			
	LFA serum IgG	RT-PCRNPOP	RT-PCR Saliva	ELISA Total Ig	LFA Serum IgG	RT-PCR NPOP	RT-PCR Saliva	ELISA Total Ig
LFA full blood IgG	**0.91**			**0.73**	**0.93**			**0.75**
LFA full blood IgM	0.30			0.33	0.19			0.25
LFA serum IgG				**0.69**				**0.82**
FISH full blood		-0.05	0.19			-0.03	-0.02	
FISH COV19_saliva		0.03	0.07			-0.03	-0.01	
FISH COV19G_saliva		0.002	0.02			-0.01	0.06	
RT-PCR_NPOP swab			0.20				0.28	

Correlations (day 0 with day 0 results per test and day 14 with day 14 results per test) considered reasonable (0.6–0.8) or good (>0.8) are presented in bold. FISH: fluorescence *in situ* hybridization; IgG; Immunoglobulin G; IgM: immunoglobulin M; LFA: lateral flow assay; N: number; NPOP: nasopharyngeal & oropharyngeal swab; PCR: polymerase chain reaction; RT-PCR: reverse transcription polymerase chain reaction.

The overall positive PCR NPOP and saliva results at day 0 correlated up to ‘reasonable’ with positive ELISA results at day 14, with Kappa values of 0.54 and 0.63 respectively. This correlation was lower for the LFA results: 0.55/0.57 (serum/full blood) and 0.62/0.64 (serum/full blood) respectively. The overall kappa correlation between the total Ig ELISA results at day 0 and 14 was good (0.84) and for the LFA-IgG in serum and LFA-IgG full blood it was reasonable (0.74 and 0.69 respectively). None of the FISH test results at day 0 correlated as ‘reasonable’ or ‘good’ with the LFA or ELISA responses at day 14.

### Interpretation of combinations of test results

Cross tabulation calculations resulted in 54 persons having a positive ELISA test outcome at day 0 and/or day 14, while having a negative PCR (n = 345) result at both day 0 and day 14, which indicates past infection. Of the PCR positive participants on day 0 (N = 59) n = 23 tested positive on ELISA at day 0, indicating ongoing infection at a late stage. Almost all PCR positive participants at day 0 (N = 59) tested PCR positive at day 14 (n = 36), indicating ongoing infection at an acute stage. When PCR and ELISA results at day 0 and 14 were combined, a total of 59 tested PCR positive and 77 tested ELISA positive.

**Fig 1:**
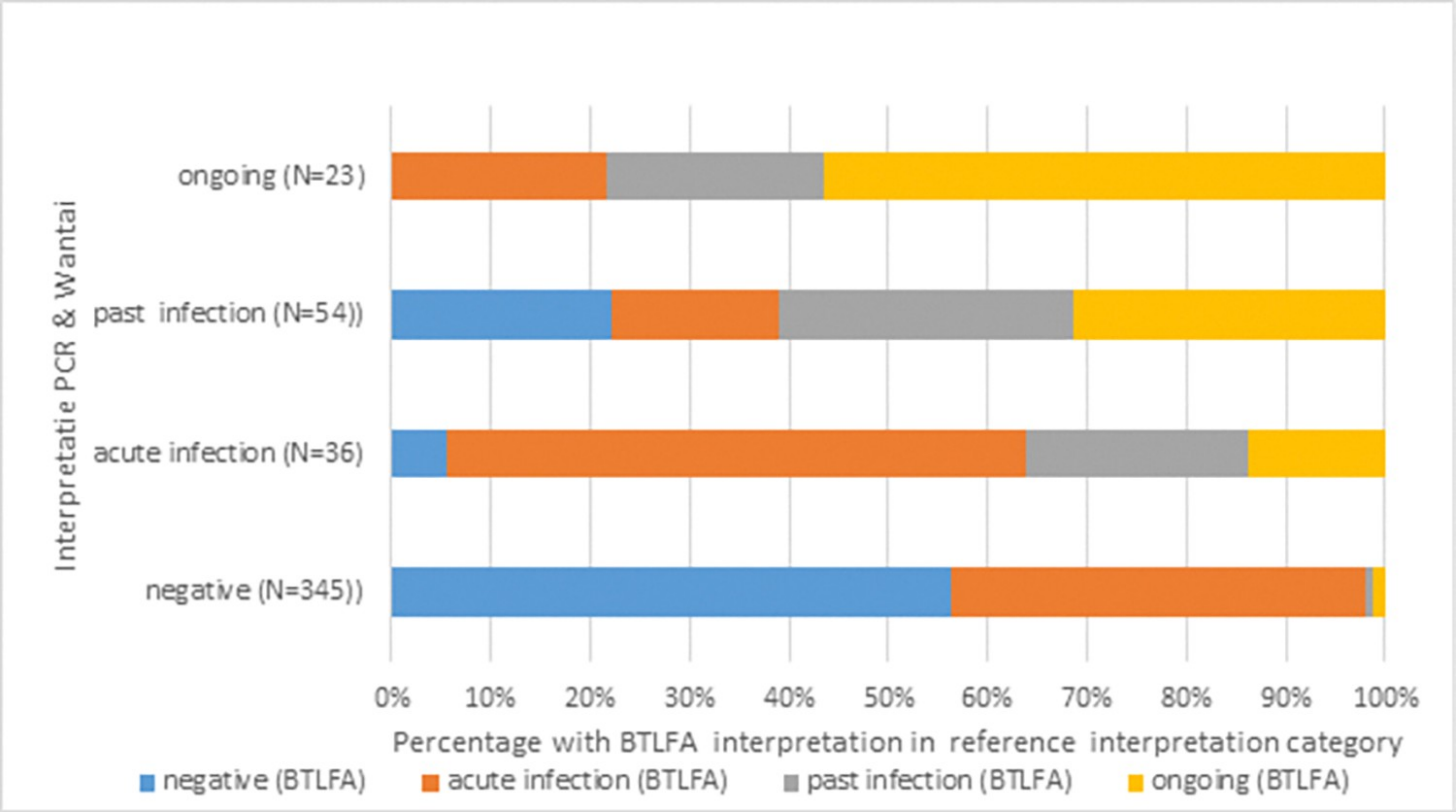
Cross tabulation of all test results. BT: FISH based analysis on any sample; LFA; Lateral flow assay on any sample.

Test results (in combination with clinical signs) should enable the medical staff to make a decision on the state of infection regarding military members of personnel individually and within the military population. The authors with a medical background therefore interpreted the results of both the outcome of the combination of PCR and ELISA test results—as well as the FISH and LFA results—for ongoing infection, past infection, acute infection or absence of infection as described in the M&M section. Clinical signs were looked at simultaneously to check whether lab results seemed biologically logical. The results are shown in Fig 1.

A comparison is shown of how the interpretation on the basis of reference lab results for any RT-PCR and ELISA result compared with those based on the interpretation of any FISH and LFA results combinations excluding LFA IgM results.

The outcome of the cross tabulation shows reasonable similarity. Although the highest percentages are in agreement with the reference lab outcome, there is a large deviation between negative reference test outcomes and interpretation based on BT/LFA. Past infection showed least agreement.

If on the basis of reference methods a past infection had been diagnosed, the field test results would diagnose a relatively large number of patients with an ongoing infection. This would have hypothetically led to an overestimation of potentially infectious personnel, if the field tests had been used. Or, hypothetically, a risk of spread of infection if reference test results had underestimated the status of infection, which was not supported by the outcome of the correlations of ELISA or LFA at day 14 with FISH results on day 0, as shown above.

### Sensitivity and specificity of field tests

From the analysis of the analytical sensitivity and specificity of the field tests compared with the reference tests, it became clear that the FISH field tests have low sensitivity and moderate to high specificity compared with RT-PCR, while LFA field tests have moderate sensitivity and high specificity compared with ELISA (Table 11). Sensitivity/specificity of RT-PCR on saliva at day 0 compared with RT-PCR on NP/OP was 71.4/99.5. At day 14 this was 34.1/99.7 respectively.

**Table 11:** Sensitivity and Specificity (Sens/Spec)of the field tests in comparison to reference laboratory tests at day 0 and 14.

Day 0			
	Reference method		
	RT-PCR_NPOP	RT-PCR_ saliva	ELISA Total Ig
FISH _full blood	6.5/92.6	2.9/92.2	
FISH COV19 _Saliva	7.0/95.2	9.7/95.3	
FISH COV19G_Saliva	36.7/75.1	37.8/74.9	
LFA IgG full blood			64.1/90.8
LFA IgG serum			54.3/97.8
Day 14			
	Reference method		
	RT-PCR_NPOP	RT-PCR _saliva	ELISA Total Ig
FISH _full blood	2.6/95.0	0.0/95.0	
FISH COV19 _Saliva	2.5/96.9	0.0/96.9	
FISH COV19G_Saliva	24.4/76.1	53.3/77.3	
LFA IgG full blood			80.0/90.5
LFA IgG serum			78.9/98.0

FISH: fluorescence in situ hybridization; IgG; Immunoglobulin G; LFA: lateral flow assay; NPOP: nasopharyngeal & oropharyngeal;RT-PCR: reverse transcription polymerase chain reaction.

## Discussion

This study, designed at the beginning of the SARS-CoV-2 pandemic and carried out in the first year of the pandemic, shows the results of a FISH-based DNA technology and an LFA antibody test used for the direct and indirect detection of the presence of SARS-CoV-2 in a military population with no or minor clinical signs. The aim was to determine the usefulness of the two selected fieldable test methods compared to established laboratory tests and 1) evaluate the applicability of the test methods under military settings, 2) determine which specimen type would work best, 3) assess the performance of the field tests compared to generally accepted reference method and 4) create awareness of the COVID-19 status of military personnel in the early phase of the pandemic when diagnostic testing at the national level was not available for this group.

Point 1) Both field tests could be used under military circumstances. The sensitivity of the FISH method on different sample types was not satisfactory at both time points, but higher at day 14 reaching 53%; for the LFA, the sensitivity was at most 80% at day 14, but also failed to reach the previously set WHO standard of at least 90% sensitivity (see M&M).

Point 2) Saliva was shown to have potential as a specimen type for the FISH-method (Table 9) on account of the presence of an adequate number of cells, and can be obtained non-invasively with less risk of exposure to medical personnel [18]. This is an advantage over NP/OP swab or blood sampling. Saliva was the preferred FISH sample type over full blood and NP/OP swab samples. That SARS-COV-2 detection through the use of RT-PCR on saliva leads to good results has already been demonstrated in other research [19]. Since at the time of our study saliva was a new sample type, sensitivity and specificity were calculated for RT-PCR on saliva, as well with RT-PCR on NP/OP as gold standard. Especially at day 14, with a sensitivity of 34.1%, it failed to reach the WHO standard. Our data are in agreement with other studies that showed comparable sensitivities for RT-PCR results on saliva samples. A faster drop in viral load in saliva samples than in NP samples at day 14 was suggested as a cause [31, 32]. The LFA performed well for IgG on both serum and full blood, which means that fingerprick testing as an indicator of antibody presence as a result of past or ongoing infection seems feasible.

Point 3) the combined usage of the LFA and FISH tests showed variable concordance with the reference laboratory tests (Fig 1) but seems to have led to overestimation of the presence of viral material. This can result in potential overdiagnosis of acute infections on the basis of the field test outcomes. Or, alternatively, it would suggest that FISH can detect RNA where the RT-PCR has become negative or is not yet positive. However, the absence of antibody responses in the study groups in FISH positive and RT-PCR negative patients did not support the latter hypothetical explanation.

Point 4) Group 4 participants showing the highest scores for clinical signs, including the highest percentage of anosmia/ageusia, had the highest odds for a positive RT-PCR test, as expected on the basis of the case definition. Furthermore, at day 0 many participants already had an antibody response, suggesting previous exposure and infection. The study was designed in, and planned for, the first half of 2020; however, due to the efforts of the military staff taking part in this study in providing civilian medical support, the original time schedule of the study was extended. This made the possibility of previous exposure to the virus during the pandemic a rather logical possibility. On the basis of RT-PCR results, many study participants had not cleared the infection by day 14 or became positive later than day 0.

One of the lessons learned was that the production and collection of saliva required supervision, since sample contamination with e.g. food/drink remnants negatively influenced FISH results in our study. Research on saliva or sputum as sample types for COVID-19 laboratory diagnostics was being published progressively as time passed during the pandemic, indicating that the methodology of saliva production and collection was influencing outcomes (e.g. mouth washes or abstaining from food intake a certain time period before sampling) [32, 33]. Spitting or coughing seems to decrease sensitivity and salivation is preferred [19]. Others also mentioned experiencing challenges with volume and obtaining pure saliva [31, 34]. This information was not yet available in March–May 2020, nor was it subsequently taken into account in the methodology. In retrospect, supervision of saliva sample production may have increased sample quality and thus could have improved FISH results. Full blood appeared to be a useful sample type for detecting IgG using the LFA antibody test selected (Table 9), but discrimination between IgG and IgM detection can be discounted due to the much lower correlations with the ELISA results with LFA IgG or IgM alone (Table 10). This is in agreement with other studies reporting similarly [35, 36]: finding the LFA not useful for IgM- but sensitive to IgG detection. Blood can be obtained using the finger prick method, but unfortunately the FISH method did not perform well on blood samples in the study setting; this excludes combining the FISH method and LFA method using only one finger prick sample session. The virus did not seem to specifically target peripheral blood mononuclear cells, resulting in sparsity of detectable SARS-COV-2 RNA in peripheral blood cells, similar to other studies [37, 38]. This phenomenon might have contributed to the low correlations of FISH tests on blood and RT-PCR tests on NP/OP or between FISH tests on full blood at day 0 and ELISA tests at day 14.

The government policy at the time of the study was that isolation was required in the case of clinical signs and a positive SARS-COV-2 test. The clinical signs used as case definition to direct active testing, as communicated by the government, were therefore used in this study as part of the inclusion criteria for the symptomatic group. Initially, data on the effect of (alternative) testing on different sample types, or on correlations between tests and clinical signs at different time points within one individual, were limited and thus part of this study. In a later phase of the pandemic, more information on this topic became available [20, 39–43]. In many medical tests, RT-PCR has been also been shown to suffer from false negative results [44–48]. Studies on different sample types (e.g. anal swabs, saliva, blood, NP/OP swab) show that sample types affect outcomes, which is related to differences in the stage of disease, viral load and viral clearing patterns [31, 49]. PCR tests can–on the other hand–remain positive, up to 100 days after infection in specific situations [50, 51] without patients being infectious. A more realistic reason that the FISH method correlated poorly with the reference methods in this R&D phase may have been that the AI programme of the Biotrack system was not optimally trained in this phase. This is supported by the fact that further development resulted in good correlations with PCR performed on saliva [27] although the study group differed. The number of positive samples that could be supplied for training the AI programme in 2020 was rather low and did not include all phases of infection. This was mainly caused by the fact that, due to non-pharmaceutical measures to limit SARS-CoV-2 spread, new cases could only be included sporadically in the study; from these cases specimens were made available for the AI training of FISH.

Studies showed that RT-PCR on nasopharyngeal swabs were 20–30% more positive > 21 days after disease onset, compared to RT-PCR on saliva. This suggested that through the use of nasopharyngeal swabs acute infections and past infections were detected, while saliva is more likely to detect an infection at an earlier stage [47, 52]. Other studies showed positive saliva samples, where PCR was initially negative [52]. Sub-genomic messenger RNAs targeted by certain PCR assay and not by others are less likely to be detected after 5 days, whereas genomic RNA can be detected for a longer period of time, especially in sputum samples [40].

In a military setting, clinical signs are the first and possibly the only indicator that an infection has potentially been introduced within a certain population. Reporting clinical signs can trigger isolation measures in a military compound. Field testing may be the only option available and PCR testing may only be available at a much later stage. Readily available diagnostic imaging techniques or virus neutralization tests are unlikely. If targeted tests become available, there is always a risk of false negative or false positive results and commanders should be aware of that. It is advisable to use a combination of different test methods and sample types if the presence of the pathogen within a population is indicated when taking clinical signs into account [53, 54]. Test results should ideally inform the medical military community on either protection against clinical disease or infectiousness to others. Reliable information on active infection, past infection or evidence of exposure (e.g. finding genetic material of exotic pathogens) can be relevant [5] also in relation to bio-threat circumstances [55]. By using the fieldable tests, a larger proportion of the tested population would have been subject to isolation, which would be positive for limiting the spread of the disease, but negative for the number of personnel that could continue to work in close contact settings.

### Limitations of the study

There were several limitations of the study:1) NP/OP samples of the proof of principle experiments contained insufficient cell material making these unsuitable for FISH analysis, which resulted in inability to carry out FISH analyses of these samples. 2) Small numbers of positive samples could be used for training the FISH algorithm with regard to positive or negative interpretation due to limited availability of high quality reference samples originating from the reference laboratory. These samples needed to be tested for negatively of other relevant viral materials during a pandemic outbreak. This governmental reference laboratory was deeply involved in the national outbreak control and thus laboratory capabilities were limited, in the available time frame. A relatively low number training samples, potentially influences the final outcomes of the study.3) The adequate sample size, as determined in the a-priori power analysis, was not reached for all testing groups due to several, mainly logistical, reasons and priority setting by military personnel, rendering the strength of conclusions low. 4) The outcomes were influenced by the predominantly negative test results. 5) Even in Group 3, clinical signs were recorded by the time samples were taken due to the delay between inclusion in the study and the sampling time point. This delay resulted from the logistical burden on the medical staff, that was also providing civilian medical support at the same time. The medical staff would ask explicitly for health complaints, which may have changed the perception of health complaints, creating a bias towards over-reporting signs. 6) No viral cultures supported the test results. However, in routine test facilities culturing is no longer part of the diagnostic process. Moreover, outbreak control measures taken by the Dutch government were based on clinical signs in combination with antigen self-tests outcomes that had become available (and had a variable sensitivity and specificity [43]. I It would nevertheless have been interesting in order to determine infectiousness of personnel. RT-PCR Ct values have been considered a proxy for contagiousness [56] and help in the interpretation of results, but analysis of Ct value data is beyond the scope of this study. 7) ELISA includes total Ig, which could have influenced the correlation of ELISA results with LFA that identified IgG and IgM separately.

### Clinical relevance and conclusions

Both alternative, fieldable test methods appeared to be applicable in a military laboratory. The development of the FISH-based method described in this study was triggered because military personnel could not be tested for SARS-CoV-2 infection at the beginning of the pandemic. Testing was needed in order to be able to contain the infection while continuing military tasks. The study shows that implementing an at that time newly developed fieldable testing is feasible in an early outbreak situation and that the system was applicable in a military environment. The results of the study also indicated the need to optimize the methodology, especially the sampling methods of the preferred sample type saliva. Following this study, a reduction in the volumes of consumables and a reduction of the FISH hybridization incubation period have been implemented, leading to a reduction of the logistical footprint. Improvements in image quality and sampling protocols have in the meantime led to higher analytical sensitivity and specificity on saliva samples using RT-PCR as gold standard in a different population [27]. The sensitivity of both fieldable tests were below the values advised by the WHO, even though the LFA assay used was found to be reliable and applicable for IgG detection in full blood, making it practical in a military setting. This supports the doctrine that field testing is considered as initial preliminary identification, while a second confirmation in a reference laboratory remains the standard in accordance with the North Atlantic Treaty Organisation’s principles regarding bio-identification and monitoring methods.
