# Correction: The performance of a lateral flow SARS-CoV-2 antibody assay and semi-autonomous SARS-CoV-2 antisense and sense RNA fluorescence in situ hybridization assay in a prospective cohort pilot study within a Dutch military population

**DOI:** 10.1371/journal.pone.0320389

**Published:** 2025-03-10

**Authors:** Inge D. Wijnberg, Anton J. Soons, Johan G. Reimerink, Marit Wiersma, Marie Christine J. Plat, Tom van Gool, Gijsbert J. Jansen, Cornelis Stijnis, Jack G. Koning, Adam Meijer

The affiliation for the third author is incorrect. Johan G. Reimerink is not affiliated with #3 but with #9: National Institute for Public Health and the Environment (RIVM), Centre for Infectious Diseases Research, Diagnostics and Laboratory Surveillance, Bilthoven, NLD
